# Energy Metabolism in *Mycobacterium gilvum* PYR-GCK: Insights from Transcript Expression Analyses Following Two States of Induction

**DOI:** 10.1371/journal.pone.0099464

**Published:** 2014-06-13

**Authors:** Abimbola Comfort Badejo, Won Hyong Chung, Nam Shin Kim, Jin Choul Chai, Young Seek Lee, Kyoung Hwa Jung, Hyo Joon Kim, Young Gyu Chai

**Affiliations:** 1 Department of Molecular and Life Science, Hanyang University, Ansan, Korea; 2 Korean Bioinformation Center, Korea Research Institute of Bioscience and Biotechnology, Daejeon, Korea; 3 Department of Nanobiotechnology, Hanyang University, Seoul, Korea; University of Padova, Medical School, Italy

## Abstract

*Mycobacterium gilvum* PYR-GCK, a pyrene degrading bacterium, has been the subject of functional studies aimed at elucidating mechanisms related to its outstanding pollutant bioremediation/biodegradation activities. Several studies have investigated energy production and conservation in *Mycobacterium*, however, they all focused on the pathogenic strains using their various hosts as induction sources. To gain greater insight into *Mycobacterium* energy metabolism, mRNA expression studies focused on respiratory functions were performed under two different conditions using the toxic pollutant pyrene as a test substrate and glucose as a control substrate. This was done using two transcriptomic techniques: global transcriptomic RNA-sequencing and quantitative Real-Time PCR. Growth in the presence of pyrene resulted in upregulated expression of genes associated with limited oxygen or anaerobiosis in *M.gilvum* PYR-GCK. Upregulated genes included succinate dehydrogenases, nitrite reductase and various electron donors including formate dehydrogenases, fumarate reductases and NADH dehydrogenases. Oxidative phosphorylation genes (with respiratory chain complexes I, III –V) were expressed at low levels compared to the genes coding for the second molecular complex in the bacterial respiratory chain (fumarate reductase); which is highly functional during microaerophilic or anaerobic bacterial growth. This study reveals a molecular adaptation to a hypoxic mode of respiration during aerobic pyrene degradation. This is likely the result of a cellular oxygen shortage resulting from exhaustion of the oxygenase enzymes required for these degradation activities in *M.gilvum* PYR-GCK.

## Introduction

Respiration is a fundamental process in all living organisms, whether aerobic or anaerobic. The basic process of respiration involves three major steps which include: (1) donation of electrons by a low-redox potential electron donor (e.g. NADH, FADH_2_), (2) electron transfer via a range of membrane-associated redox co-factors or complexes, and (3) the reduction of a high redox potential electron acceptor (oxygen, oxyanions of metals, transition metals and inorganic elements), thereby terminating the process. This “electron transfer” or respiratory chain is situated inside the mitochondrial membrane or cell membrane of eukaryotes and prokaryotes, respectively. Cellular energy, synthesized and released in the form of ATP, is produced from an electrochemical gradient (proton-motive force) generated via these electron transfer processes.

Extreme respiratory flexibility exists in bacteria because they have a vast range of electron acceptors, conferring upon them the ability to colonize many of earth's habitats including the most hostile micro-oxic and anoxic environments [1]. In mycobacteria, this flexible respiratory ability has been reported [2] and attributed to the presence of genes responsible for ATP generation by oxidative phosphorylation (Ubiquinone cytochrome b reductase complex and cytochrome c oxidase) and to genes encoding anaerobic terminal electron acceptors such as nitrate reductase, fumarate reductase and nitrite reductase. This group of aerobes is unique in that they have characteristically strong cell envelopes which give them the ability to survive in stressful environments. In some pathogenic mycobacterial species, the cell wall is reported to aid in protective invasion of their hosts [3] and resistance to antibiotics [4]. Some non-pathogenic strains also perform unique activities including biodegradation and bioremediation of toxic pollutants [5].

*Mycobacterium gilvum* PYR-GCK was isolated from the sediment of the Grand Calumet River in Northwestern Indiana based on its ability to utilize pyrene, a toxic polycyclic hydrocarbon, as a growth substrate [6]. Many reports list the presence of atmospheric oxygen as important for hydrocarbon degradation while others have demonstrated the possibility of anaerobic hydrocarbon degradation. Widdel and Rabbus [7] showed that the biochemical mechanisms of aerobic and anaerobic hydrocarbon degradation are completely different. *M.gilvum* PYR-GCK has been cultivated aerobically in studies evaluating the molecular events occurring during pyrene degradation and in studies aimed at the development of more effective bioremediation strategies [8], [9]. The results revealed links between metabolic pathways and respiratory mechanisms. In the current study, a more in-depth analysis of the respiratory activities of *M.gilvum* PYR-GCK was initiated, using pyrene and glucose as test and control substrates respectively. We utilized a gene expression study to evaluate genetic biomarkers for respiratory processes in *M.gilvum* PYR-GCK. Previous studies on mycobacterial respiratory pathways have been published. Ortega-Calvo and Gschwend [10] reported that sorption to sediment black carbon resulted in oxygen limitation for aerobic Polycyclic Aromatic Hydrocarbon (PAH) biodegradation. Furthermore, Fritzsche [11] reported that pyrene degradation at low oxygen concentrations does not produce any additional metabolites or intermediates which might be expected as the result of the activity of an oxygenase with low oxygen affinity, such as aromatic ring cleavage monooxygenases. With the high affinity of the aromatic ring cleavage dioxygenases for molecular oxygen, there is the possibility of more oxygen being diverted for dioxygenase activity as compared to cytochrome oxidase activity. The aim of the present investigation was to determine the molecular basis of this shift in respiration based on the expression of various respiratory enzyme components measured in a constantly aerated culture medium.

## Materials and Methods

### Reagents and bacterial strains

*Mycobacterium gilvum* PYR-GCK was acquired from the American Type Culture Collection (ATCC) under the code name *M. flavescens* ATCC 700033. The strain was maintained in Bacto Brain Heart Infusion Media (BD Laboratories, USA) at 29°C or as a stock preserved in the same media supplemented with 28% glycerol and stored at –80°C. The pyrene substrate (confirmed >98.0% pure by Aldrich Company) and other chemicals used were purchased from Sigma-Aldrich Company (St. Louis, USA) and Tokyo Chemical Industry (Tokyo, Japan).

### Media and cultivation

*M. gilvum* PYR-GCK cells were grown in 500 ml flasks containing 200 ml basal medium containing, per litre: NaNO_3_, 0.5 g; (NH_4_)_2_SO_4_, 1.0 g; Na_2_HPO_4_; 2.5 g; KH_2_PO_4_, 1.0 g; MgSO_4_•7H_2_O, 0.1 g; Fe(NH_4_)_2_(SO_4_)_2_, 5 mg; 1 ml filter-sterilized vitamin solution (containing, per litre: p-aminobenzoic acid, 200 mg; biotin, 200 mg; folic acid, 200 mg; nicotinic acid, 200 mg; Ca-panthothenate, 100 mg; pyridoxine-HCl, 100 mg; riboflavin, 100 mg; thiamine, 100 mg and vitamin B12, 1 mg) and 1 ml Trace Elements solution (containing, per litre: MnCl_2_•2H_2_O, 23 mg; H_3_BO_3_, 31 mg; CoCl_2_•6H_2_O, 36 mg; CuCl_2_•2H_2_O, 10 mg; NiCl_2_•6H_2_O, 20 mg; ZnCl_2_, 50 mg and Na_2_MoO_4_•2H_2_O, 30 mg) sterilized separately. Pyrene was dissolved in acetone and added to a flask of prepared minimal media at a final concentration of 50 µM while a separate minimal media flask was supplemented with 0.38 g/L _D_-glucose from a sterile stock. The culture media was adjusted to pH 7.5. Cells were harvested from Bacto Brain Heart Infusion agar plates and washed twice in 50 mM monobasic sodium phosphate buffer. A 15 ml sample of cells, with an absorbance of OD_545_ 2.956 per 1 ml, was inoculated into a culture flask containing 105 ml of 50 mM monobasic sodium phosphate buffer without any carbon source and incubated for at least 4 hours at 30°C, as suggested by Betts et al. [12]. After 6 hours, the sodium phosphate buffer culture was divided into six parts and used to inoculate 200 ml minimal basal medium supplemented with either pyrene or glucose, in triplicate. To ensure the possible identification of expressed genes during the different stages of the metabolism process, cultures were further induced at 24 and 48 hours through the repeated addition of the substrates from sterile stocks. The inoculated culture flasks were incubated at 30°C for 50 hours in the dark with shaking at 150 rpm.

### Total RNA extraction and purification

Total RNA was extracted as reported in Badejo et al. [8], with slight modifications applied to the harvested bacterial cells. In summary, RNAprotect Bacteria Reagent (Qiagen, USA) was added to the culture broth of 50-hour-old bacterial cells in a 2µ1 ratio. Cells were then harvested from all six bacterial cultures by centrifugation at 10,000× *g* at 4°C for 1 min. RNAiso (Takara, Japan) lysing solution was added to the cells, along with 10 µl β-mercaptoethanol and 0.6 g of 0.1 mm Zirconia/Silica beads (BioSpec, USA). The mix was processed in a mini Bead-beater (BioSpec, USA) for 45 seconds and immediately placed on ice. Two hundred microliters of chloroform was added to the solution and the tubes were gently inverted for 5 min to mix. The tubes were then centrifuged at 12,000× *g* for 15 min at 4°C and the clear top layer was gently transferred to a new tube. Five hundred microliters of isopropanol was added and the tubes were gently inverted to mix once again before a final incubation on ice for 1 hour. After incubation, the lysed mix was centrifuged at 12,000 × *g* for 10 min at 4°C and the isopropanol was discarded. Ice-cold 70% ethanol was added to the RNA pellet to gently wash. Following another round of centrifugation as above, the ethanol was carefully removed. The pellets were then left to dry at room temperature for 3–5 minutes before reconstitution in 20 µl RNase-free water. RNA was treated with RNase-free DNase (Promega, USA) to remove residual DNA and then purified using RNeasy MinElute Cleanup Kit (Qiagen, USA). The quantity and quality of the purified RNA was determined using Agilent 2100 Bioanalyzer (Agilent Technologies). Samples with an RNA Integrity number (RIN) value ≥8 [13] were aliquoted for use in separate analyses.

### Global transcriptome analysis using RNA sequencing

RNA processing and sequencing: A set of triplicate total RNA samples isolated from bacteria under common induction conditions were combined (to avoid biased results), purified and sequenced at Macrogen Korea using the Illumina Genome Analyzer. Prior to sequencing, total RNA was processed using a Ribo-Zero rRNA removal kit (Epicentre Biotechnologies, Madison, USA) to remove the 23 S and 16 S ribosomal RNAs. Enrichment was assessed using Agilent 2100 Bioanalyzer and RNA 6000 chip (Agilent Technologies). Remaining mRNA was fragmented into small pieces using divalent cations under elevated temperature and converted into a library of template molecules suitable for subsequent cluster generation using the reagents provided in the Illumina TruSeq, RNA Sample Preparation Kit (Illumina Inc., USA), following the manufacturer's instructions. The cleaved RNA fragments were copied into first strand cDNA using reverse transcriptase and random primers. Second strand cDNA was then synthesized using DNA polymerase I and RNase H. The cDNA fragments were then processed through an end repair reaction by the addition of a single ‘A’ base, followed by ligation of the adapters. The products of these reactions were then purified and enriched with PCR to create the final cDNA library. The cDNA fragments were sequenced with HiSeq2000 (Illumina Inc. USA).

Alignment and filtering of sequence reads: The alignment and sequenced fragment reads bioinformatics processing was done at the Korean Bioinformation Center (KOBIC), Daejeon. A total of 40,012,820 and 21,440,720 fragments were generated from the glucose-induced and pyrene-induced RNA samples, respectively. Differential gene and transcript expression analyses of the sequence reads were performed with TopHat and Cufflinks. Data was normalized by calculating the “fragments per kilo base per million map reads” (FPKM) for each gene [14]. Briefly, data was treated with low-quality filtering and reads were removed before CuffDiff analysis. Exact duplicated reads were removed using Prinseq version 0.19.3, with average quality ≥Q20. Filtered reads were mapped to the reference genome [Genbank CP000656, CP000657, CP000658, and CP000659] using Bowtie2 version 2.0.0, default option [15]. Expression levels of the chromosome and plasmids were analyzed separately. Afterwards, differential expression analysis was performed using Cuffdiff (default option) from the Cufflinks 2.0.2 package [16]. Results were manually curated to identify pyrene degradation related transcripts by comparing pyrene-induced transcripts with the glucose-induced transcripts. The data acquired was deposited in the Gene Expression Omnibus data base (GEO: GSE44536).

### Gene expression analysis by quantitative Real-Time PCR

Complementary DNA preparation: Aliquots of total RNA (10 µg), isolated and purified as described earlier, were reverse transcribed, resulting in a total of six samples. The reverse transcription step was carried out using random hexamer primers and the PrimeScript 1st Strand cDNA Synthesis Kit (Takara, Japan) according to manufacturer's instructions. Briefly, random hexamers and RNA templates were mixed and denatured at 65°C for 5 min followed by cooling for 2 min on ice. 5X Primescript buffer, RTase and RNAse inhibitor were added to the cooled template mix and incubated for 1 hr at 50°C before enzyme inactivation at 70°C for 15 min. Negative control reactions lacking RTase were performed to test for the presence of genomic DNA contamination in the RNA samples.

Quantitative Real-Time PCR: Complementary DNA samples were diluted 1.5-fold and relative quantification real-time PCR was carried out in a standard fashion using SYBR Premix Ex-Taq II (Takara Bio, Otsu Shiga, Japan) according to manufacturer's directions. An AB-7500 Real-Time PCR System (Applied Biosystems Inc., California, USA) was employed for the real-time PCR analysis. Primer3 software [17] and the NCBI primer designing tool were used to design primers that would amplify a product of approximately 200 base-pairs. Amplicon expected sizes and the absence of non-specific products were confirmed by analysis of PCR products on 2% agarose gels in TAE buffer, stained with ethidium bromide and visualized under UV-light. PCR reactions were assembled according to the manufacturer's instructions, and three technical replicates for each sample were included. Twenty microliter PCR reactions contained 0.4 µM of each primer (Table 1). Each PCR analysis included a no-template control containing water instead of cDNA as well as an RT negative control for each gene. The amplification conditions were: 95°C for 15 s; 40 cycles of 95°C for 15 s and 60°C for 1 min. The specificity of the reaction was confirmed by obtaining a melting curve from 55–95°C. The efficiency of the reactions was automatically calculated by the PCR machine.

**Table 1:** List of respiratory function genes from *M.gilvum* PYR-GCK used for validation.

Gene ID	Gene Symbol	Gene	Primers
Mflv_4481	*nuoA*	NADH dehydrogenase subunit A	5′-GTACTACCTGACCGCGATGC-3′
			3′-CGTACGCATAGGCCACGAAT-5′
Mflv_4493	*nuoM*	NADH dehydrogenase subunit M	5′-CCTCCATCTCGCATTTCGGT-3′
			3′-TGGAGATGCCGTGATTGACC-5′
Mflv_0571	*sdhA/frdA*	succinate dehydrogenase/fumarate reductase subunit A	5′-AGTAACTCCAGGCAGCGAAC-3′
			3′-AGTGTCATGTCTTCACGGCG-5′
Mflv_4847	*sdhB/frdB*	succinate dehydrogenase/fumarate reductase subunit B	5′-GTACCTGGACGGCACATTGA-3′
			3′-GCTGCTTGTTCGGGTTCTTC-5′
Mflv_4844	*sdhC*	succinate dehydrogenase subunit C	5′-CATCGAGACCTACAAGACCCC-3′
			3′-CGTTGAGAGCGTGGTAGAGC-5′
Mflv_4845	*sdhD*	succinate dehydrogenase subunit D	5′-TGGCTGTTCATGCGGTTCTC-3′
			3′-GGTACACACCGTTCTCCCAC-5′
Mflv_2593	*fdhD*	formate dehydrogenase, subunit D	5′-TCGTGTAGAAGTTGCGGGTG-3′
			3′-TCGGGCTATTCGCAGAACAC-5′
Mflv_2249	*fdhD2*	formate dehydrogenase	5′-CGAAAACCCGTGATCCCCAA-3′
			3′-GTCGTCCTCCATCCCTACGA-5′
Mflv_3013	*nirB*	nitrite reductase	5′-AGTTTGTCGTAGTGCAGCGT-3′
			3′-CCTGCTGTCGAATGTGCTTG-5′
Mflv_3684	*cox15*	cytochrome C oxidase	5′-AGGTGCTGTTCTACGCCTG-3′
			3′-CACCACAGCAGACCTGTGA-5′
Mflv_5097	*rpoB*	RNA polymerase β-subunit	5′-TCTCGTGCTCTTCGATGTGG-3′
			3′-GTGGGAGGGTCACAACTACG-5′

Included are the primers used to amplify the required genes using quantitative Real-Time PCR.

### Expression analysis

Ten genes were studied using the qRT-PCR assay; two coding for subunits of the Type-1 NADH dehydrogenase (*nuoA, M*), four coding for the subunits of succinate dehydrogenase/fumarate reductase (*sdhA/frdA, sdhB/frdB, sdhC, sdhD*), one coding for a high oxygen-affinity cytochrome c oxidase (*cox15*), a nitrite reductase gene (*nirB*) and two formate dehydrogenase genes (*fdhD*, *fdhD2*). The mRNA levels of these genes were determined for six samples (two induced states in triplicate) after a 50 hour exposure to pyrene induction with the aim of evaluating which genes were up- or down-regulated as a consequence of the treatment. To analyze differential gene expression, the mRNA levels were compared between the pyrene-induced genes and the glucose-induced genes. In all cases, the expression of all genes was quantitated after normalization of their RNA levels relative to the expression of the *rpoB* gene, which codes for the β-subunit of bacterial RNA polymerase (Mflv_5097), as previously described [8]. The fold change was calculated using the relative quantification (2^−ΔΔCT^) method [18]. Statistical analysis using the Student's t-test was performed using the SPSS v. 21.0 software package for Windows (SPSS Inc., USA). When the differential *p-*value was less than 0.05, it was regarded as statistically significant.

## Results

### Summary of global transcriptome expression analyses

Global transcriptome expression profiling was carried out using RNA sequencing technology. Induced transcripts were enriched and identified as outlined in the Materials and methods section. Functional annotation of gene transcripts and gene expression profiles was performed using the bioinformatics tool, DAVID (Database for Annotation, Visualization, and Integrated Discovery) [19]. We identified 1,381 genes, representing 25.26% of the total expressed and identified gene arsenal, which were highly expressed under pyrene-induced conditions. Relative expression ratios were derived by comparing mRNA abundance levels in cells grown in pyrene substrate relative to mRNA levels in glucose grown cells. Genes displaying a two-fold or greater change in transcript abundance were considered to be up-regulated. Of the 5,613 total genes (both chromosomal and from three plasmids) in *M.gilvum* PYR-GCK, 2,597 were differentially up or down regulated by two-fold or greater. Growth on pyrene resulted in considerable changes in the transcription profile versus the glucose grown reference across all Clusters of Orthologous Groups (COGs), however the most significant changes primarily involved the genes which function in energy metabolism and pyrene-substrate metabolism (Table 2). A detailed list of energy production and conversion genes that displayed increases in their expression levels is presented in Table 3. Other genes up-regulated as a result of pyrene induction were mostly involved in carbon substrate metabolism, including glyoxalase dioxygenase genes (Mflv_0538, Mflv_0618, Mflv_1264) and muconolactone delta-isomerase (Mflv_1359). Noteworthy among the glucose-induced genes was the high expression of transcriptional and ribosomal gene constituents compared to the pyrene-induced genes (Table 2).

**Table 2:** List of the most significantly expressed transcripts based on COG (Clusters of Orthologous Groups) classification.

COG ontology	Gene count	*P*-value
**Upregulated genes**		
*Biological processes*:		
Oxidation reduction	157	5.30E-08
Aromatic compound catabolic process	35	6.00E-03
Energy production and conversion	54	5.00E-02
*Cellular components*:		
Integral to membrane	97	1.90E-06
*Molecular function*:		
Electron carrier activity	96	8.90E-08
Oxidoreductase activity	28	5.00E-04
Iron-sulfur cluster binding	33	3.80E-03
**Downregulated genes**		
*Biological processes*:		
Transcription regulation	127	3.50E-09
Fatty acid metabolic process	9	8.90E-03
Protein complex biogenesis	12	3.30E-02
*Cellular components*:		
Extracellular region	8	3.80E-02
*Molecular function*:		
Transcription regulator activity	106	5.60E-08
DNA binding	139	7.80E-03
Coenzyme binding	53	1.40E-02
Transferase activity	18	2.10E-02

The normalized data acquired from differential RNA sequence analyses of *M.gilvum* PYR-GCK was curated with the DAVID bioinformatics tool.

**Table 3:** Changes in transcript expression levels of energy metabolism genes from *M.gilvum* PYR-GCK grown in different substrates.

Gene ID	Gene Name	Relative expression
**ATP synthase**		
Mflv_2312	ATP synthase F0 subcomplex A subunit	0.118
Mflv_2313	ATP synthase F0 subcomplex C subunit	0.382
Mflv_2316	ATP synthase F1 subcomplex alpha subunit	0.990
Mflv_2315	ATP synthase F1 subcomplex delta subunit	0.795
Mflv_2317	ATP synthase F1 subcomplex gamma subunit	0.586
Mflv_2318	ATP synthase F1, beta subunit	0.880
Mflv_2319	ATP synthase F1, epsilon subunit	0.364
**Succinate dehydrogenase/Fumarate reductase**		
Mflv_0394	succinate dehydrogenase subunit A	0.847
Mflv_4846	succinate dehydrogenase subunit A	1.210
Mflv_0395	succinate dehydrogenase subunit B	0.888
Mflv_4847	succinate dehydrogenase subunit B	1.214
Mflv_4844	succinate dehydrogenase subunit C	1.800
Mflv_4845	succinate dehydrogenase subunit D	2.934
Mflv_1519	fumarate reductase/succinate dehydrogenase flavoprotein domain protein	0.717
Mflv_1547	fumarate reductase/succinate dehydrogenase flavoprotein domain protein	2.235
Mflv_0669	fumarate reductase/succinate dehydrogenase flavoprotein domain protein	-
Mflv_1621	fumarate reductase/succinate dehydrogenase flavoprotein domain protein	1.184
Mflv_4550	fumarate reductase/succinate dehydrogenase flavoprotein domain protein	1.774
Mflv_2398	fumarate reductase/succinate dehydrogenase flavoprotein domain protein	2.182
Mflv_3411	fumarate reductase/succinate dehydrogenase flavoprotein domain protein	3.169
Mflv_3157	fumarate reductase/succinate dehydrogenase flavoprotein domain protein	3.539
Mflv_4564	fumarate reductase/succinate dehydrogenase flavoprotein domain protein	4.307
Mflv_4567	fumarate reductase/succinate dehydrogenase flavoprotein domain protein	5.002
Mflv_0571	fumarate reductase/succinate dehydrogenase flavoprotein domain protein	7.201
Mflv_2403	fumarate reductase/succinate dehydrogenase flavoprotein domain protein	2.114
**Electron transfer complexes**		
Mflv_1196	Cytochrome-c oxidase	0.264
Mflv_2818	Cytochrome-c oxidase	2.952
Mflv_2949	Cytochrome c oxidase, subunit II	0.383
Mflv_4277	Cytochrome-c oxidase	0.200
Mflv_1196	Cytochrome-c oxidase	0.264
Mflv_5341	Cytochrome-c oxidase	1.694
Mflv_5377	Cytochrome-c oxidase	1.862
Mflv_2818	Cytochrome-c oxidase	2.952
Mflv_3585	cytochrome bd quinol oxidase subunit 1 apoprotein	0.038
Mflv_3586	cytochrome bd quinol oxidase subunit 2 apoprotein	0.074
Mflv_5339	cytochrome c assembly protein	2.563
Mflv_2820	cytochrome c assembly protein	4.671
Mflv_5329	cytochrome c biogenesis protein, transmembrane region	1.336
Mflv_5368	cytochrome c biogenesis protein, transmembrane region	0.806
Mflv_2825	cytochrome c biogenesis protein, transmembrane region	2.149
Mflv_0051	cytochrome c biogenesis protein, transmembrane region	2.868
Mflv_3217	cytochrome c biogenesis protein, transmembrane region	3.440
Mflv_2949	cytochrome c oxidase, subunit II	0.383
Mflv_2956	cytochrome c oxidase, subunit III	0.174
Mflv_1813	cytochrome c oxidase, subunit III	1.021
Mflv_4575	cytochrome c oxidase, subunit III	1.492
Mflv_5349	cytochrome c oxidase, subunit III	2.031
Mflv_5384	cytochrome c oxidase, subunit III	2.703
Mflv_2809	cytochrome c oxidase, subunit III	4.631
Mflv_1250	cytochrome c oxidase, subunit III	8.972
Mflv_3684	cytochrome oxidase assembly	0.277
Mflv_2953	menaquinol-cytochrome c reductase cytochrome b subunit precursor	0.532
Mflv_2955	menaquinol-cytochrome c reductase cytochrome c1 subunit precursor	0.457
Mflv_2954	menaquinol-cytochrome c reductase iron-sulfur subunit precursor	0.625
Mflv_4481	NADH dehydrogenase subunit A	8.603
Mflv_4482	NADH dehydrogenase subunit B	2.103
Mflv_4483	NADH dehydrogenase subunit C	2.107
Mflv_4484	NADH dehydrogenase subunit D	1.900
Mflv_4485	NADH dehydrogenase subunit E	2.254
Mflv_4486	NADH-quinone oxidoreductase, F subunit	1.197
Mflv_4487	NADH dehydrogenase subunit G	2.478
Mflv_4488	NADH dehydrogenase subunit H	1.742
Mflv_4489	NADH dehydrogenase subunit I	2.110
Mflv_4490	NADH dehydrogenase subunit J	2.345
Mflv_4491	NADH dehydrogenase subunit K	0.343
Mflv_4492	NADH dehydrogenase subunit L	1.817
Mflv_4493	NADH dehydrogenase subunit M	3.045
Mflv_4494	NADH dehydrogenase subunit N	0.801
Mflv_1689	NADH:flavin oxidoreductase/NADH oxidase	0.410
Mflv_4880	NADH:flavin oxidoreductase/NADH oxidase	1.555
Mflv_4553	NADH:flavin oxidoreductase/NADH oxidase	3.710
Mflv_0933	NADH:ubiquinone oxidoreductase complex I intermediate-associated protein 30	3.342

Bold numbers highlight genes differentially regulated above and below two-fold.

### Validation of changes in respiratory gene expression using quantitative RT-PCR

**Figure 1:**
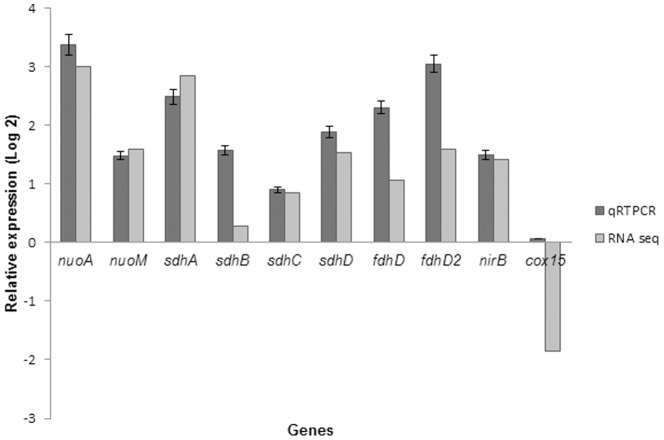
Expression patterns of *M.gilvum* PYR-GCK respiratory genes regulated by different substrates. RNA was extracted and transcripts were analyzed by RNA-sequencing and qRT-PCR. Similar expression levels were observed among the two data sets with the cox-15 gene used as a negative control value. Only those genes with p -value ≤0.05 and an expression-fold change of <0.5 and ≥2.0 are included in the plot. Error bars represent standard deviations.

In order to confirm the expression profiles obtained from the RNA-seq expression data, qRT-PCR analysis was carried out on ten genes randomly selected on the basis of their biological significance (Table 1) using total RNA isolated from exponential cultures of *M. gilvum* PYR-GCK grown separately in either glucose or pyrene. In general, the expression of most genes tested correlated strongly with the data obtained from RNA-seq (Fig. 1). The expression levels of *nuoA* (Mflv_4481), *nuoM* (Mflv_4493), *sdhA*/*frdA* (Mflv_0571), *sdhD* (Mflv_4845), fdhD (Mflv_2593), fdhD2 (Mflv_2249) and *nirB* (Mflv_3013) were found to be up-regulated in the pyrene induced cells compared to the control cells. Although the *cox-15* gene, which codes for high-oxygen affinity cytochrome-c oxidase (Mflv_3684), was found to be down-regulated in the RNA-seq data (0.277 fold), qRT-PCR analysis revealed a normal expression level (1.05 fold), as was the case for *sdhB*/*frdB* (Mflv_4847) and *sdhC* (Mflv_4844). Mflv_5097 (DNA-directed RNA polymerase subunit: *rpoB*) was used as an internal control to calculate the fold-change between the pyrene and glucose-induced states.

## Discussion

Microbial growth requires the biosynthesis of a specific range of monomers which are assembled into polymers to form the bulk of new biomass [20], [21]. Microorganisms aerobically take up different carbon compounds as carbon and energy sources, and degrade them into intermediates which are then utilized in the central metabolic pathway. Energy production in a living cell is intertwined with these metabolic processes as they require the input of energy and precursors in various forms (NADPH, ATP, PEP, acetyl CoA, transmembrane proton gradient, etc.). In aerobic respiration when glucose is available as a substrate, most of the free energy released during the oxidation of glucose to CO_2_ is retained in the reduced coenzymes NADH and FADH_2_ which are generated during glycolysis and the citric acid cycle. The electrons released from these reduced coenzymes are eventually transferred to O_2_ to form H_2_O according to the following strongly exergonic reactions: 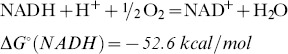





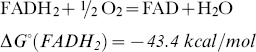



In pyrene metabolism, the metabolites enter into the central metabolic pathway via acetyl CoA and the tricarboxylic acid cycle (TCA) or gluconeogenetic pathway. This process skips the glycolytic-sourced NADH contributed to the electron carriers. Hence, the respiratory chain is left with electron carriers sourced mainly from the TCA cycle, NADH (from isocitrate oxidation, oxidative decarboxylation of alpha ketoglutarate and malate oxidation) and FADH_2_ (from succinate oxidation), and other preparatory reactions. NADH generates protons across the membrane while FADH_2_ does not due to its insufficient energy for proton pumping. For this reason, FADH_2_ has limited function in ATP production. In our study, the use of pyrene as a sole substrate resulted in very significant changes in gene expression relative to growth with glucose, with the majority of the genes involved in energy metabolism being affected (Table 3). As expected, most of the affected genes included oxidoreductases and genes with functions within the respiratory chain.

**Figure 2:**
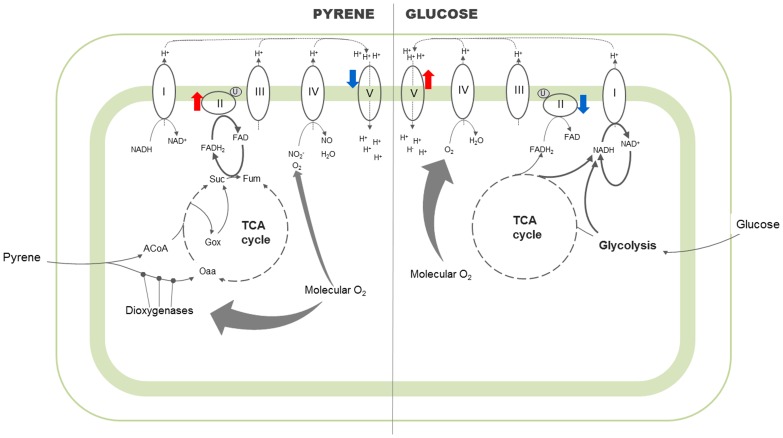
Proposed comparative scheme of bioenergetic pathways operative in *M. gilvum* PYR-GCK grown in different substrate conditions. Only major pathways based on our gene expression results are shown, with bold arrows designating the significant pathways. The intramembranous electron transfer pathways are not shown for the sake of simplicity. Dioxygenase activities within the pyrene degradation pathway are denoted with 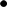. The roman numerals correspond to the five complexes of the oxidative phosphorylation pathway (I - NADH dehydrogenase, II - Fumarate reductase, III - Cytochrome bc complex, IV - Cytochrome oxidase, V - ATP synthase), with discussed complexes marked with colored arrows (red for upregulated- and blue for down regulated- gene transcripts respectively). Abbreviations: ACoA – Acetyl CoA; Oaa – Oxaloacetate; Gox – Glyoxalate; Suc – Succinate; Fum – Fumarate, O_2_ – Oxygen; U – Ubiquinone; NADH – reduced Nicotinamide adenine dinucleotide, NAD^+^ - oxidized Nicotinamide adenine dinucleotide, FADH_2_ – reduced Flavin adenine dinucleotide; FAD^+^ - oxidized Flavin adenine dinucleotide; NO_2_^−^ - nitrite; NO-Nitrous oxide; H_2_O – water; H^+^ - hydrogen ions.

The use of FADH_2_ in energy production (fumarate respiration) is considered a common pathway for energy metabolism in adaptation to hypoxic environments in bacteria [22]. FADH_2_ is transferred to a low potential quinone, such as naphthoquinone, by complex I and is finally oxidized by the fumarate reductase activity of complex II which is a reverse reaction of the succinate–ubiquinone reductase (SQR) activity of complex II. By using this respiratory chain, bacteria are able to synthesize ATP even in the absence of oxygen. FADH_2_ is recycled within complex II of the respiratory chain and the succinate oxidation step of the TCA cycle [23] is catalyzed by the succinate dehydrogenase (*sdhA-D*) and fumarate reductase (*frdA-D*) enzymes (Fig 2). In our research, we demonstrated strong upregulation of these genes in pyrene-fed cells as compared to the glucose-fed control cells (Table 3). Omura et al.[24] and Piedad et al. [25] reported the possible sourcing of succinate via the phosphoenolpyruvate carboxykinase pathway (PEPCK), which occurs in microaerophilic or hypoxic conditions, leading to the production of FADH_2_ for respiration and energy production. Our earlier report [9] on pyrene metabolism showed upregulated protein expression of the enzymes involved in the carboxylation of PEP to oxaloacetate (OAA). This process which reverses the normal glycolytic activity of pyruvate kinase may be recruited into the PEPCK-succinate pathway, via OAA and malate, to function as a source of the highly regulated *sdh*/*fdr* genes and resulting in the strong activity of complex II on the respiratory chain (Fig 2).

The glyoxalate shunt has been reported to be activated as a result of cellular oxygen shortage [26]. This shunt results in increased production of succinate and malate from isocitrate, thereby skipping three oxidation steps (isocitrate oxidation, oxidative decarboxylation of alpha ketoglutarate and malate oxidation) in order to conserve energy in microaerophilic conditions. Although the genes functioning in this shunt were not significantly affected in the current study, our previous proteomic study revealed that isocitrate lyase is a highly regulated enzyme. The shunt is also utilized under anaerobic conditions and again results in the exclusion of three oxidation steps, however, we observed upregulation of many type 1-NADH dehydrogenase subunit genes (*nuoA-C, E-F, I-J, M*) as well as the upregulation of eight fumarate reductase genes. In all states of respiration, nicotinamide adenine dinucleotide (NAD) generation is crucial for energy production. Frezza et al. [27] determined the state of mitochondrial NAD in a hypoxic state using a metabolomic study of the ratio between oxidized and reduced NAD molecules. Our results were based solely on transcriptomics techniques, however, we believe further studies utilizing techniques such as metabolomics and mineralization studies will reveal more about the adaptive techniques of the strain.

In strong aerobic conditions, especially during vigorous metabolic activities, the activities of numerous genes in the respiratory pathway including complexes I, III, IV and V have been reported to be upregulated, as aerobic organisms will preferably opt for respiratory pathways with greater energy production output. This may be the reason why the glucose fed cells did not display high levels of expression of the complex 2 genes. Finally, as a support to our observed results of uprgeulated type-1 NADH dehydrogenase genes, upregulated activity of formate dehydrogenase genes were also observed in both gene expression results. Formate dehydrogenase functions in anaerobic nitrate respiration by forming a complex with lipid soluble quinone [28]. Nitrate and nitrite reductase genes are known to function in bacterial anaerobic respiration. Although the nitrate reductase genes were not significantly upregulated in our study, nitrite reductase was upregulated. The upregulated expression of these genes may be as a result of formate produced from aromatic substrate metabolism rather than by fermentation as reported by Ferry and Wolfe [29].

Since pyrene was degraded aerobically with the metabolites and respective gene products confirmed in previous studies [6], [9], [30], the microaerophilic condition in the pyrene-induced bacterial cells might have been a result of oxygenase activities. Numerous mono- and dioxygenases are very active during the degradation of aromatic compounds [31]; and these oxidoreductases incorporate oxygen atoms from molecular oxygen (O_2_) into their substrates. These important enzymes cleave the ultra-stable aromatic ring structures in the notoriously hard-to-degrade polycyclic aromatic hydrocarbon pollutants in the environment.

## Conclusions

We have examined cellular respiration in two bacterial induction conditions; using pyrene (similar to bacterial toxic polycyclic aromatic hydrocarbon degradation) and glucose as test and control samples, respectively. The interesting results observed focused on a probable microaerophilic respiratory activity during a fully-aerobic pyrene biodegrading activity. These observations were supported by gene expression results from two different analyses. Consequently, we suggest that despite the availability of ample molecular oxygen from culture aeration, the metabolizing cell must have undergone cellular-molecular oxygen shortage. This was likely due to the activity of the oxygenase genes which resulted in oxygen depletion during the pyrene degradation pathway activities.
